# Do “brassy” sounding musical instruments need increased safe distancing requirements to minimize the spread of COVID-19?a)

**DOI:** 10.1121/10.0002182

**Published:** 2020-10-15

**Authors:** Thomas R. Moore, Ashley E. Cannaday

**Affiliations:** Department of Physics, Rollins College, Box 2743, Winter Park, Florida 32789, USA

## Abstract

Brass wind instruments with long sections of cylindrical pipe, such as trumpets and trombones, sound “brassy” when played at a fortissimo level due to the generation of a shock front in the instrument. It has been suggested that these shock fronts may increase the spread of COVID-19 by propelling respiratory particles containing the SARS-CoV-2 virus several meters due to particle entrainment in the low pressure area behind the shocks. To determine the likelihood of this occurring, fluorescent particles, ranging in size from 10–50 μm, were dropped into the shock regions produced by a trombone, a trumpet, and a shock tube. Preliminary results indicate that propagation of small airborne particles by the shock fronts radiating from brass wind instruments is unlikely.

## I. INTRODUCTION

The current Centers for Disease Control guidelines recommend maintaining a distance of approximately 2 m between people to reduce the spread of SARS-CoV-2, the virus that is responsible for COVID-19.^1^ This distance is based on the estimate that the mean distance exhaled droplets will travel during normal conversation is approximately 1 m. However, there are circumstances where droplets will carry much further. One obvious case of extended droplet travel occurs when a person sneezes, in which case the expelled droplets can travel as far as 8 m.^2^ Unfortunately, there are many situations where the appropriate limit for physical distancing to reduce the spread of the virus is unknown. This lack of knowledge may lead to an increase in spreading the disease as the restrictions on human interactions are reduced in an effort to enhance economic activity, but it may also lead to unnecessary restrictions on activities that do not contribute to the pandemic.

In many regions of the world the entertainment industry is a major contributor to the local economy, and as the entertainment industry begins to function again one of the primary beneficiaries will be musicians. Although musicians are critical to the economies of many communities, there appears to be little research into the requirements for effective distancing from musical instruments to reduce the spread of disease. In many cases, the requirement of maintaining approximately 2 m between people will likely be adequate. However, the amount of air exhaled from brass instruments, combined with the known nonlinear effects that cause wave steepening, indicates the possibility that some members of the brass wind family of instruments have the potential to spread an airborne virus significantly farther. Additionally, it is possible that exhaled droplets emitted by the musician or anyone in the vicinity of the instrument may be propagated much further than a few meters due to the shock wave exiting the bell of these instruments, as suggested by recent studies on particle entrainment in shock waves.^3^

It has been known for many years that when brass wind instruments with long cylindrical sections of pipe are played at a high volume, a shock wave is created inside the instrument that is subsequently radiated into the air. This shock wave is responsible for the “brassy” sound produced by trumpets and trombones. The presence of the shock wave from these instruments has been verified by direct observation,^4^ and the theory that describes their origin is well understood.^5,6^ It has also recently been shown that small particles are accelerated by a passing shock front. This acceleration does not occur because the shock front pushes the particle as one may suspect, but because small particles can be entrained into a low pressure area behind the shock front.^3^ The acceleration can be quite large, on the order of 10^7^ m/s^2^, with terminal velocities exceeding 150 m/s for shock fronts produced by Mach numbers as small as 1.2.

In the event of a single passing shock front, the displacement of a particle will be on the order of millimeters. However, in the case of brass wind instruments, the shock wave is not a single event. Rather, it is a series of shock fronts occurring hundreds or thousands of times per second. Under these conditions an estimated travel of several meters is not unreasonable, given that it has been shown that even weak spherical shock fronts can persist several hundred meters from the source.^7^ Therefore, it is plausible that the distances that exhaled droplets travel in the presence of loud brass music is much greater than 2 m.

It is also known that large water droplets can be broken into smaller droplets when a shock wave passes.^8^ These smaller droplets may persist in the air for longer times than millimeter-sized airborne droplets containing SARS-CoV-2 that may be exhaled by an infected individual when coughing or sneezing. The possible reduction in droplet size may result in an increase in the number of infected droplets being propagated long distances by subsequent shock fronts, adding to the importance of determining whether the shock fronts from brass instruments can, indeed, contribute to spreading of COVID-19.

## II. EXPERIMENTS AND RESULTS

**FIG. 1.**
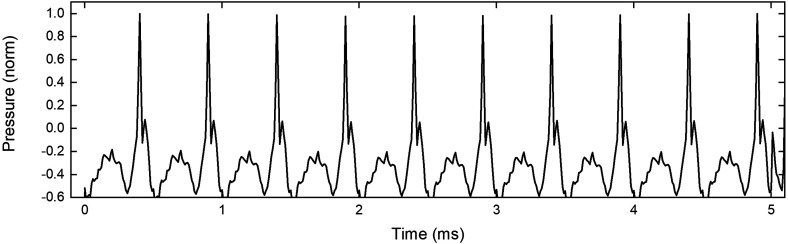
Radiated waveform from a trombone at a frequency of 2 kHz. The SPL was approximately 117 dB measured 1 m from the bell.

To determine if it is necessary to revise the physical distancing requirements for trumpets and trombones during performance, we attached a compression driver to the mouthpiece of a trombone with the slide fully extended. The extension of the slide ensured that the maximum cylindrical tubing was available to create the shock front. The input to the trombone mouthpiece was sinusoidal, and the frequency was varied between 500 Hz and 2.5 kHz, with the sound pressure level (SPL) measured 1 m from the bell varying from approximately 110 dB at 500 Hz to approximately 119 dB at 2.5 kHz. The radiated pressure at 2 kHz is shown as a function of time in Fig. 1, demonstrating that the slope of the wave fronts was significantly increased by transmission through the instrument.

The axis of the trombone was oriented parallel to the floor, with the center of the bell approximately 80 cm above it. The bell and floor area were enclosed in a wooden box to ensure that ambient air currents did not affect the results. The floor was covered in black paper. Fluorescent chalk dust was dropped approximately 10 cm in front of the bell to simulate the presence of small aspirated particles. The particles were dropped from approximately 20 cm above the bell, both by hand and through a piece of filter paper with an approximately 1 mm diameter hole. The method of dropping the particles did not affect the results.

The sizes of the particles were measured on the floor after the experiment using a microscope with a calibrated reticle. The smallest particles were measured to be 10 ± 3 *μ*m. In addition to the chalk dust, strontium aluminate crystals impregnated with fluorescent dye were used to study the effects on larger particles. The particle sizes of the crystals were stated by the manufacturer as ranging from 15–50 μm; however, we found no crystals smaller than 20 ± 3 μm. It is believed that the transmission of COVID-19 occurs primarily through drops with diameter greater than 5 μm;^9^ therefore, it is not unreasonable to use chalk dust and strontium aluminate crystals to simulate the disease carrying particulate, provided the difference in mass is taken into account.

Strontium aluminate and chalk dust both have sizes commensurate with aspirant particles; however, aspirant droplets are largely made of water, and the densities of both are significantly different than water. The specific gravity of strontium aluminate is approximately 3.5 and chalk dust has a specific gravity of approximately 2.5. The difference in mass between these substances and water droplets will affect the time the particle is suspended in the air as well as any acceleration by the shock front. The time that the particles are suspended in the air is a function of the atmospheric drag, which, given the sizes of the particles, is assumed to be linear. Assuming a spherical particle, the terminal velocity is approximately proportional to the density. The horizontal distance traveled is also linearly proportional to the density. Therefore, we assume that any asymmetric propagation observed due to the shock front must be multiplied by the square of the specific gravity to approximate the result achieved for an aspirant particle. This is likely an overestimation due to the non-spherical nature of chalk and strontium aluminate crystals, but a factor of 12 is probably a reasonable upper bound.

**FIG. 2.**
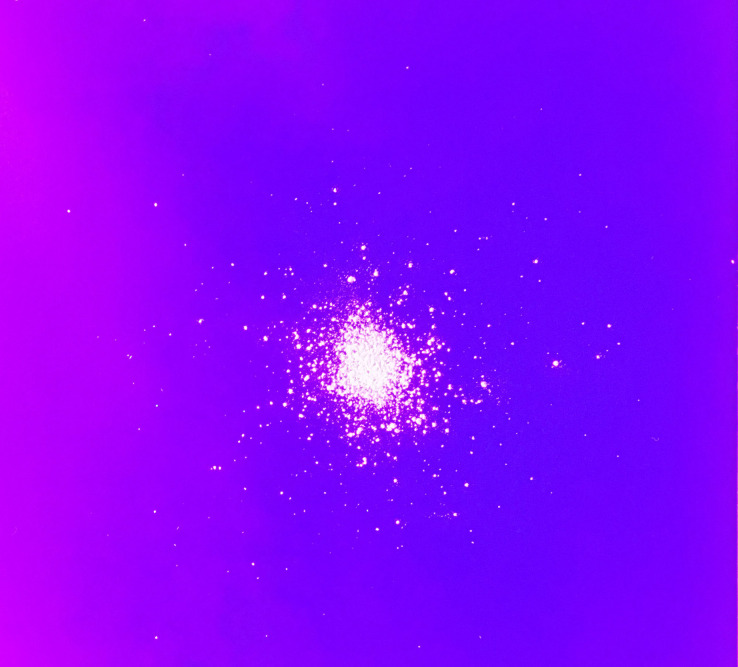
(Color online) Pattern of chalk dust on the floor below the trombone bell. The particles extend approximately 10 cm from the center of the pattern. There is no evidence of asymmetric propagation.

The chalk dust and crystals were each dropped while the signal from the compression driver produced oscillations in the air column within the trombone. The compression driver was driven by a sine wave from a function generator, and the frequency was varied by hand between 100 Hz and 2.5 kHz while observing the falling particles. Additionally, the signal was held constant at 500 Hz, and 1, 2 and 2.5 kHz for several seconds. Following the experiments, the paper under the trombone bell was examined using an ultraviolet light to determine the extent of the dispersion of the particles. The ultraviolet light caused the particles to fluoresce, allowing easy identification as well as the ability to distinguish the particles of interest from dust and dirt. A typical dispersion pattern of the chalk dust is shown in Fig. 2. The pattern of the strontium aluminate crystals was similar.

Although there was some small dispersion of the particles below the bell, resulting from random drift attributable to the non-spherical nature of the particles, and possibly some slight initial off-axis velocity of the particles when they were dropped, it was consistent with what was observed when particles were dropped in front of the bell without the shock fronts present. The maximum extent of the particles from the center of the pattern was approximately 10 cm in both cases, representing a symmetrical 6° cone from the point at which they were dropped. In no case did the pattern exhibit a measurable preference for directionality. Similar experiments with a trumpet produced the same result.

The waveform shown in Fig. 1 clearly shows steepening similar to that observed during play. However, the waveforms from a trombone played by an experienced musician that are shown in Fig. 2 of Hirschberg *et al.*^5^ indicate that the secondary pressure maxima between shock fronts are not commonly produced during performance. The enhanced steepening of the wave shown in Hirschberg *et al.*^5^ is likely due to the non-sinusoidal input waveform that is produced by lips, which is different from the sinusoidal input in the experiments described here. The result of the differing input is that the radiated waveform shown in Fig. 1 is not identical to that produced by an experienced musician.

**FIG. 3.**
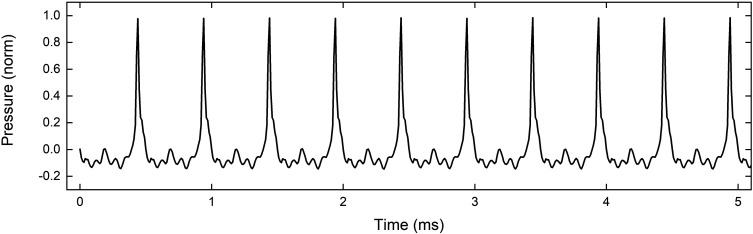
Radiated waveform from cylindrical pipe at a frequency of 2 kHz. The SPL was approximately 107 dB measured 1 m from the end of the pipe.

To produce a shock front more similar to that produced during musical performance, the experiments described above were repeated with the compression driver attached to a 3.1 m long cylindrical pipe. Although the input signal is different from that produced by lips, and the bore shape is different from a trombone, the radiated waveform is similar. The waveform from the cylindrical pipe produced by a 2 kHz input sine wave is shown in Fig. 3, demonstrating enhanced wave steepening and a single pulse per cycle, similar to that in Fig. 2 of Hirschberg *et al.*^5^ and Fig. 6 of Thompson and Strong.^6^

The pipe was 25.4 mm in diameter and the shock front was driven by the compression driver at several frequencies between 1 and 2.5 kHz. The SPL measured 1 m from the end of the pipe was approximately 107 dB between 1 and 2 kHz, and 112 dB at 2.5 kHz.

The results of the experiments using the shock tube were similar to the results found when the trombone was used. We could find no difference between the dispersion pattern of the particles on the floor in front of the pipe when the shock wave was present and when there was no sound. It is interesting to note that when chalk dust was placed inside the pipe near the open end, particles were expelled from the pipe when the driving frequency matched an acoustic resonance of the pipe, as one might expect. At resonance frequencies above 1 kHz a significant amount of chalk dust was expelled, but the forward motion was not maintained after leaving the pipe. Typically, the chalk dust stopped its horizontal motion within approximately 5 cm and fell onto the floor without traveling any farther.

## III. ANALYSIS AND CONCLUSIONS

The lack of propagation of small particles by the shock front is probably due to the fact that the shock is relatively weak. It is proposed by Prestridge^3^ that the high accelerations and velocities of small particles that were observed by shock fronts created at Mach 1.2 are attributable to the disturbance in the air caused by the wake of the particle after the shock passes. This disturbance creates a low pressure area that accelerates the particle into the flow that follows the shock front. However, in the case of brass wind instruments, Pandya *et al.*^4^ suggest that for a trumpet played at a fundamental frequency of approximately 700 Hz by an experienced musician the shock Mach number is on the order of 1.00007. We are not aware of any other measurement to confirm that this is a representative value for all brass wind instruments, but if it is, it would not be surprising that the disturbance created by the passing of the shock front around a particle would be small.

It will be necessary to complete several experiments with professional musicians under controlled conditions to conclusively demonstrate that there is no significant propagation of small particles due to the shock fronts produced by cylindrical brass wind instruments during performance. The role of the air flow exiting the instrument and how it interacts with the shock front is also unknown and needs to be carefully investigated, as does the appropriateness of applying the theory proposed by Prestridge^3^ to periodic shock fronts with Mach numbers very close to one.

The results of these experiments indicate that the danger of spreading SARS-CoV-2 by brass wind musicians due to the presence of the radiated shock front is probably minimal. Further research is required to conclusively demonstrate this is true during performance; however, based on the results of the experiments described here, we find no justification for imposing more stringent distancing requirements for brass wind players simply due to the presence of the shock waves produced during loud play. To our knowledge, however, the appropriate distancing requirements that will reduce the spread of COVID-19 has yet to be determined for any musical instrument.

## References

[1] Centers for Disease Control website available at https://www.cdc.gov/coronavirus/2019-ncov/prevent-getting-sick/prevention.html (Last viewed July 27, 2020).

[2] L. Bourouiba , “ Turbulent gas clouds and respiratory pathogen emissions: Potential implications for reducing transmission of COVID-19,” JAMA 323, 1837–1838 (2020). 10.1001/jama.2020.4756 32215590

[3] K. Prestridge , “ Drag of shock-accelerated microparticles,” in *WIT Transactions on Engineering Sciences* ( WIT, Southampton, 2019), Vol. 123, pp. 3–9.

[4] B. H. Pandya , G. S. Settles , and J. D. Miller , “ Schlieren imaging of shock waves from a trumpet,” J. Acoust. Soc. Am. 114, 3363–3367 (2003). 10.1121/1.1628682 14714816

[5] A. Hirschberg , J. Gilbert , R. Msallam , and A. P. J. Wijnands , “ Shock waves in trombones,” J. Acoust. Soc. Am. 99, 1754–1758 (1996). 10.1121/1.414698

[6] M. W. Thompson and W. J. Strong , “ Inclusion of wave steepening in a frequency-domain model of trombone sound production,” J. Acoust. Soc. Am. 110, 556–562 (2001). 10.1121/1.1371759

[7] S. M. Young , K. L. Gee , T. B. Neilsen , and K. M. Leete , “ Outdoor measurements of spherical acoustic shock decay,” J. Acoust. Soc. Am. Exp. Lett. 138, EL305–310 (2015). 10.1121/1.4929928 26428831

[8] S. V. Poplavski , A. V. Minakov , A. A. Shebeleva , and V. M. Boyko , “ On the interaction of water droplet with a shock wave: Experiment and numerical simulation,” Int. J. Multiphase Flow 127, 103327 (2020). 10.1016/j.ijmultiphaseflow.2020.103273

[9] T. M. Cook , “ Personal protective equipment during the coronavirus disease (COVID) 2019 pandemic – a narrative review,” Anaesthesia 75, 920–927 (2020). 10.1111/anae.15071 32246849

